# Association between *Helicobacter pylori* infection and kidney damage in patients with peptic ulcer

**DOI:** 10.1080/0886022X.2019.1683029

**Published:** 2019-11-22

**Authors:** Wei Pan, Hui Zhang, Li Wang, Tingting Zhu, Bo Chen, Junming Fan

**Affiliations:** a Department of Nephrology, The Affiliated Traditional Chinese Medicine Hospital of Southwest Medical University, Luzhou, China;; b Department of Nephrology, The Affiliated Hospital of Southwest Medical University, Luzhou, China;; c Laboratory of Organ Fibrosis Prophylaxis and Treatment by Combine Traditional Chinese and Western Medicine, Research Center of Combine Traditional Chinese and Western Medicine, Affiliated Traditional Medicine Hospital of Southwest Medical University, Luzhou, China;; d The Second People's Hospital of Yibin, Yinbin, China;; e Chengdu Medical College, Chengdu, China

**Keywords:** Albumin–creatinine ratio, *Helicobacter pylori* infection, kidney damage, peptic ulcer

## Abstract

**Background:** *Helicobacter pylori* (*H. pylori*) is relevant to several renal diseases. Our previous research indicates that cytotoxin-associated gene A (CagA) of *H. pylori* increases secretion of serum immunoglobulin A1 (IgA1) and induces the underglycosylation of IgA1, one of the key factors causing IgA nephropathy. Here, we aimed to study the correlation between *H. pylori* infection and kidney damage in patients with peptic ulcer, and evaluate the effect of *H. pylori* eradication on kidney damage.

**Methods:** ^14^C-urea breath test and rapid urease tests were applied to *H. pylori* infection detection. Random urine samples are subjected to the albumin–creatinine ratio (ACR) examination. The correlation between ACR and *H. pylori* infection was analyzed in patients with peptic ulcer and healthy controls. The levels of IgA and underglycosylated IgA1 in serum are also detected by enzyme-linked immunosorbent assay (ELISA) and Helix aspersa lectin (HAA) binding assay.

**Results:** (1) *H. pylori* infection rate in patients with peptic ulcer (88.14%) is significantly higher than that in healthy controls (42.68%). (2) There is a positive correlation between *H. pylori* infection and ACR abnormal in patients with peptic ulcer (*p* = .025), while showing a negative correlation in healthy individuals (*p* = .571). (3) Urinary ACR was uncorrelated with the severity of *H. pylori* infection in the 27 abnormal urinary ACR cases of the patients with peptic ulcer. (4) After *H. pylori* eradication, the ACR rates of *H. pylori*-positive patients with peptic ulcer were significantly decreased (*p*<.01).

**Conclusions:** (1) For the *H. pylori-*positive patients with peptic ulcer, *H. pylori* infection may be a risk factor resulting in kidney damage. (2) *H. pylori* eradication probably benefits to kidney damage relief and chronic kidney disease prevention.

## Introduction

*Helicobacter pylori* (*H. pylori*) is a gram-negative microaerophilic bacterium colonized in gastrointestinal tract, which is regard as common alimentary bacteria resulting into chronic infection. Once infected by *H. pylori*, it is difficult for patients to eradicate this bacterium through innate immunity, which will cause many chronic diseases. Increasing evidence supports *H. pylori* infection inducing a range of gastrointestinal symptoms in patients [1]. Other than gastrointestinal diseases, the infection with *H. pylori* also involves into the occurrence and process of cardiovascular diseases, respiratory diseases, hematological diseases, metabolic dysfunction diseases, urogenital diseases, skin diseases, etc. [2,3]. However, the underlying mechanisms are poorly understood. It is demonstrated that the specific cytotoxin of *H. pylori* or/and inflammatory response caused by *H. pylori* maybe the reason that *H. pylori* infection could induce these non-gastrointestinal diseases [4,5].

*H. pylori* infection may also involve into the occurrence of renal diseases. It has been reported that *H. pylori* is relevant to several renal diseases such as diabetic nephropathy, membranous nephropathy, Henoch–Schonlein purpura nephritis, immunoglobulin A (IgA) nephropathy, etc. [6]. *H. pylori* antigen can be found in the pathological tissues in renal diseases [7]. Our previous basic research indicates that cytotoxin-associated gene A (CagA) of *H. pylori* increases secretion of serum immunoglobulin A1 (IgA1) and induces the underglycosylation of IgA1 through promoting the proliferation of B lymphocytes [8], in which underglycosylated IgA1 is well known as one of the key factors causing IgA nephropathy. A clinical prospective study also suggests the possibility of *H. pylori* infection triggering renal diseases [9].

However, the correlation between *H. pylori* infection and kidney damage in healthy population is less studied. Besides, large sample size is required and more accurate indicators should be evaluated to elucidate the relationship between *H. pylori* infection and kidney damage in patients with gastrointestinal diseases. In this study, *H. pylori* infection rates were examined in patients with peptic ulcer diagnosed endoscopic examination and matched healthy controls. The correlation between kidney damage and *H. pylori* infection in patients with peptic ulcer and healthy controls was also investigated in this study.

## Materials and methods

### Patients and methods

Healthy controls consisting of 390 individuals (aged 18–60 years) were recruited from Physical Examination Center, The Affiliated Traditional Chinese Medicine Hospital of Southwest Medical University. Exclusion criteria for healthy controls included systolic blood pressure ≥ 140 mmHg, diastolic blood pressure ≥90 mmHg, LEU leucocyte ≥1+, blood leucocyte >10 × 10^9^/L, fasting blood-glucose ≥ 7.0 mmol/L, aspartate aminotransferase > 40 U/L, glutamic transaminase > 40 U/L, creatinine > 133 μmoI/L, BMI ≥ 30 kg/m^2^ or with a history of diabetes mellitus, liver and kidney diseases such as liver cirrhosis or renal calculus, etc. Subjects consisting of 194 patients (aged 18–60 years) with peptic ulcer were recruited from gastroscopy room, Gastroenterology, Affiliated Hospital of Southwest Medical University. Exclusion criteria for patients with peptic ulcer included (1) systolic blood pressure ≥ 140 mmHg or diastolic blood pressure ≥ 90 mmHg, (2) recent infection such as respiratory infection, urinary tract infection, etc., (3) proceeding anti-*H. pylori* therapy or taking PPI or H2 receptor blockers or antibiotics in recent four weeks, and (4) a history of cardiovascular disease, liver and kidney disease, hematological system diseases, endocrine diseases, rheumatic immune system diseases, etc.

From September 2014 to August 2015, 390 physical examination population were recruited to the study. One hundred and seventy-one patients who were suffering from dyspeptic complaints and who had no exclusion criteria were recruited to the study. At this time, written informed consents were obtained from all the patients. According to power analysis [10], *α* = 0.05 and *β* = 0.20; the power of the study was 99.1%.

### Identification of *H. pylori* infection

^14^C-urea breath test was applied for *H. pylori* infection detection in healthy controls and rapid urease tests were used for *H. pylori* infection examination in patients with peptic ulcer.

*^14^C-urea breath test*: According to the manufacturer’s instruction (Anhui Jinren Medical Instrument Co., Ltd., Anqing, China), after at least three hours fasting, subjects swallowed the ^14^C-urea capsule (1 μCi) with 30 mL lukewarm water, and sit-ins 15 min. Then subjects exhaled to a ‘Breath Test Card’ for breath samples collection. When the displayer of the ‘Breath Test Card’ turns blue to white, it suggested the completion of samples collection. The ‘Breath Test Card’ was analyzed by the ^14^C-Urea Breath Test Machine. When the number of disintegrations per minute (dpm) >99.0, *H. pylori* infection was identified as positive. On the other side, when dpm ≤ 99.0, *H. pylori* infection was identified as negative.

*Rapid urease tests*: The antrum gastric mucosa biopsy samples from patients with peptic ulcer were obtained for *H. pylori* colonization analysis using a PYLORI-TEST paper (Zhuhai Kedi Technology Co., Ltd., Zhuhai, China). According to the instruction of this rapid urease test kit, the test paper turned red suggesting the positive *H. pylori* infection, while no color changed indicating negative. By referring to the research of Ghasemi Basir et al. [11], we used the Sydney system grading of chronic gastritis for grading of *H. pylori* density [12]. Scattered organisms covering less than one third of the surface are regarded as mild colonization (*H. pylori* (+)); large clusters or a continuous layer over two-thirds of the surface is graded as severe (*H. pylori* (+++)); intermediate numbers are mentioned as moderate colonization (*H. pylori* (++)).

### Urine ACR test

The urine albumin–creatinine ratio (ACR) is widely used to evaluate kidney damage. Both the levels of albumin and creatinine were measured in random urine samples, and then the ACR was calculated. The urine albumin was measured by cerebrospinal fluid (CSF) and urine protein assay kit (Siemens Healthcare Diagnostics Inc., Tarrytown, NY). The urine creatinine was measured by Creatinine assay kit (Rongsheng-biotech Company, Shanghai, China).

### Enzyme-linked immunosorbent assay

Serum samples were obtained from healthy controls in Physical Examination Center. Blood was drawn into tubes without any anticoagulant in the morning after an overnight fast and the tubes were left in a standing position for about 30 min at room temperature, then centrifuged at 25 °C, 1500×*g* for 10 min. Subsequently, the serum was subjected to enzyme-linked immunosorbent assay (ELISA) and Helix aspersa lectin (HAA) assay or stored at –80 °C.

IgA level in serum was measured using ELISA. Briefly, 96-well plates were coated with IgA primary antibody (Southern Biotechnology Associates, Birmingham, AL**)** overnight at 4 °C. Then serum samples were added to the plates, and incubated overnight at 4 °C. After washed by PBS thrice, plates were incubated with secondary antibody (Southern Biotechnology Associates, Birmingham, AL). Tetramethyl benzidine (TMB) dilution was used for color development, and OD value was measured with Microplate Reader (Bio-Rad, Hercules, CA) at 450 nm.

### Helix aspersa lectin binding assay

Helix aspersa lectin binding assay was applied to determine the underglycosylation of IgA1 in serum. Serum samples were added to the IgA antibody-coated 96-well plates, and the captured IgA1 was treated with neuraminidase for 3 h. Subsequently, biotinylated HAA lectin was added to the plates for 3 h incubation at 37 °C. Avidin-horseradish peroxidase conjugate was used for lectin binding. The results were measured as above.

### *H. pylori* eradication therapy

Proton pump inhibitor-based quadruple therapy was performed in patients with peptic ulcer and *H. pylori* infection, including 1000 mg amoxicillin twice daily, 20 mg lansoprazole twice daily, 500 mg clarithromycin twice daily, and 220 mg bismuth subcitrate twice daily for 14 days. The efficacy of eradication was modified using the urea breath test.

### Statistical analysis

Statistical analyses were performed using the SPSS 17.0 statistical software program (SPSS Inc., Chicago, IL). The data were shown as the means ± standard deviations (SDs). Chi-square test and Fisher’s exact test were used to analyze the difference among groups. Spearman’s rank correlation coefficient was applied to evaluate the correlation between *H. pylori* infection severity and abnormal ACR rate. *p* Values<.05 denoted a statistically significant difference.

## Results

### *H. pylori* infection rates

The healthy control subjects, totally 390 individuals including 98 females and 292 males, were performed ^14^C-urea breath test for *H. pylori* infection determination. The results showed the number of infected individuals was 161 including 39 females and 122 males, with the total infection rate 41.28% (161/390), including 39.80% (39/98) in female and 41.78% (122/292) in male (Table 1). There was no significant difference in *H. pylori* infection rate between females and males.

**Table 1.** *H. pylori* infection rate in the two groups.

Variable	Healthy controls (*n* = 390)	Patients with peptic ulcer (*n* = 194)	*t*/*χ*^2^	*p*
Gender (M/F)	292/98	123/71	–	–
Age (years)	45.09 ± 9.19	46.07 ± 9.62	*t*=–1.191	.234
Fasting blood-glucose (mmol/L)	5.15 ± 0.48	5.16 ± 0.46	*t*=–0.252	.801
Systolic BP (mmHg)	119.08 ± 6.42	119.48 ± 5.84	*t*=–0.749	.455
Diastolic BP (mmHg)	69.98 ± 5.03	69.69 ± 5.53	*t* = 0.615	.539
BMI (kg/m²)	25.1 ± 3.2	25.7 ± 3.0	*t*=–1.204	.229
ALT (U/mL)	13.88 ± 5.78	14.43 ± 5.97	*t*=–1.077	.582
AST (U/mL)	15.81 ± 5.00	15.47 ± 5.71	*t* = 0.703	.483
*H. pylori* infection rate (M/F)	41.28% (41.78%/39.8%)	88.14% (89.43%/39.80%)	*χ*^2^=115.98	<.001

BP: blood pressure; BMI: body mass index; ALT: alanine transaminase; AST: aspartate transaminase.

The patients with peptic ulcer, 194 individuals in total consisted of 71 females and 123 males, were performed rapid urease tests for *H. pylori* infection detection. The results showed the total infection rate was 88.14% (171/194) within 85.92% (61/71) in female and 89.43% (110/123) in male (Table 1). There was no significant difference in *H. pylori* infection rate between females and males.

**Figure 1.**
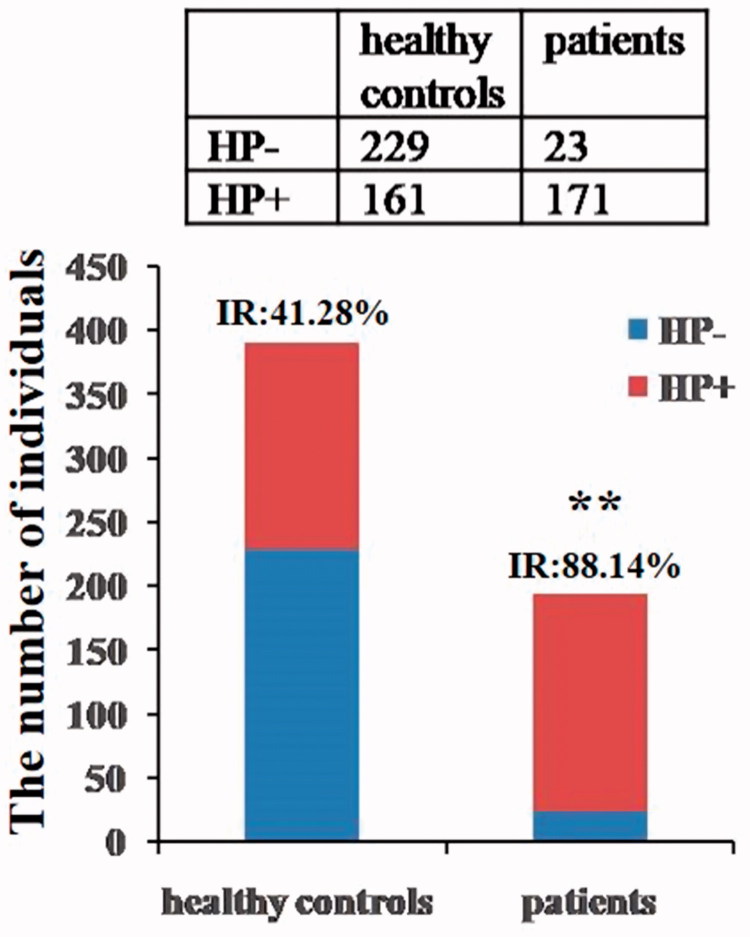
*H. pylori*-negative and *H. pylori*-positive subjects in two groups. Both ^14^C-urea breath test and rapid urease tests were utilized for identifying *H. pylori* infection in healthy controls and patients with peptic ulcer (IR: infection rate, **p<.01 vs. healthy control).

Compared with healthy control group, the *H. pylori* infection rate in patients with peptic ulcer was highly increased (*χ*^2^ = 115.98, *p*<.001) (Figure 1), and no significant difference in *H. pylori* infection rate was found between females and males in both groups.

*H. pylori* infection is probably a crucial factor for the occurrence of gastrointestinal diseases, which will activate the inflammatory response in individuals. The release of inflammatory cytokines has a toxic effect on kidney, and long-term chronic inflammation always leads to the kidney damage, companied by abnormal urine ACR. Therefore, whether *H. pylori* infection is associated with kidney damage was investigated next in healthy individuals and patients with peptic ulcer.

### Correlation between *H. pylori* and kidney damage in healthy control group

The urine ACR was examined to evaluate the kidney damage. In healthy controls, the abnormal urine ACR was 7.95% (31/390), including 6.83% (11/161) in *H. pylori* infected individuals and 8.73% (20/229) in *H. pylori* uninfected individuals (Table 2). Urine ACR data analysis showed no statistical difference between *H. pylori*-negative and *H. pylori*-positive individuals (*χ*^2^ = 0.467, *p* = .571) in healthy group.

**Table 2.** The *H. pylori* infection and urine ACR in healthy controls.

Groups	ACR normal	ACR abnormal	Abnormal rates
*H.pylori*negative	209 (13.11 ± 5.32)	20 (49.74 ± 26.07)	8.73% (20/229)
*H. pylori*positive	150 (13.48 ± 6.94)	11 (55.57 ± 21.91)	6.83% (11/161)
*χ*^2^			0.467
*p*			.571

Besides, the levels of serum IgA1 and underglycosylated IgA1, the specific biomarkers of IgA nephropathy, were also tested by ELISA and HAA assay. Fourteen *H. pylori*-negative and 16 *H. pylori*-positive individuals were randomly chosen from healthy controls for serum IgA and underglycosylated IgA1 detection. However, both the serum IgA and underglycosylated IgA1 levels showed no significant difference between *H. pylori*-negative group and *H. pylori*-positive group.

### Correlation between *H. pylori* and kidney damage in patients with peptic ulcer

In the group of patients with peptic ulcer, the abnormal urine ACR rate was 13.92% (27/194), including 15.79% (27/171) in *H. pylori*-positive individuals and 0% (0/23) in *H. pylori*-negative individuals (Table 3). All the 27 individuals with abnormal urine ACR were determined as *H. pylori* positive and all the 23 *H. pylori*-negative individuals presented normal urine ACR. The abnormal rate of ACR in *H. pylori*-positive group showed a significant difference compared to *H. pylori*-negative group through Fisher's exact test (*p* = .040). The results suggested that *H. pylori* infection was probably an important risk factor for kidney damage in patients with peptic ulcer.

**Table 3.** The urinary ACR and *H. pylori* infection in patients with peptic ulcer.

Groups	Urine ACR normal	Urine ACR abnormal	Abnormal rates
*H. pylori*negative	23	0	0% (0/23)
*H. pylori* positive	144	27	15.79% (27/171)
*p*			.040

Moreover, we tried to analyze the correlation between *H. pylori* colonization severity and urine ACR. The *H. pylori* colonization severity was defined as mild colonization (*H. pylori* (+)), moderate colonization (*H. pylori* (++)), severe (*H. pylori (+++)*) based on the rapid urease tests. However, the results from 27 *H. pylori* infection with abnormal urine ACR demonstrated no positive correlation (*r_s_* = –0.198, *p* = .323) (Table 4).

**Table 4.** The *H. pylori* infection severity level and urinary ACR abnormal severity.

Infection severity	Number	ACR (mg/g)
*H. pylori* (+)	9	106.90 ± 111.54
*H. pylori* (++)	15	93.03 ± 169.70
*H.pylori* (+++)	3	57.40 ± 21.86
*r_s_*		–0.198
*p*		.323

### Follow-up study on patients with gastrointestinal diseases after *H. pylori* eradication

**Figure 2.**
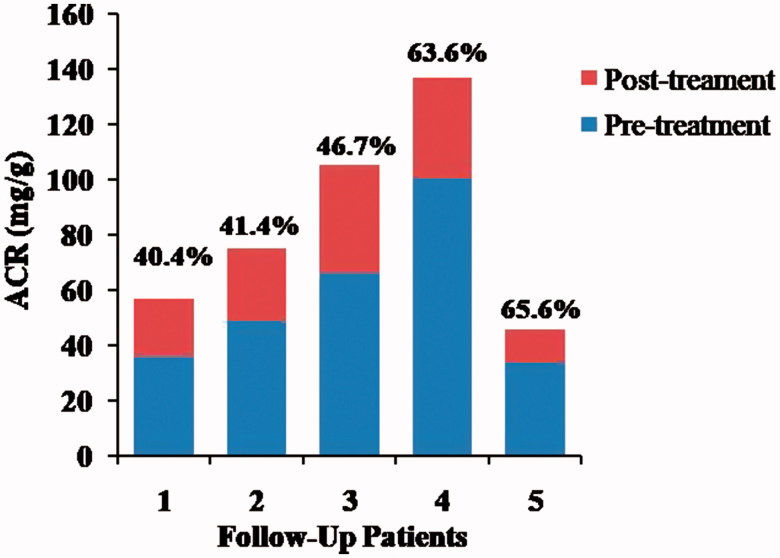
The urinary ACR between pretreatment and post-treatment in *H. pylori* infection patients with peptic ulcer. ACR of five subjects was significantly decreased after 3 month routine *H. pylori* eradication therapy by urine ACR test.

In order to elucidate the relationship between *H. pylori* infection and kidney damage, we tried the follow-up study on *H. pylori* eradiation. Five patients with peptic ulcer received 3 month routine medical treatments for the *H. pylori* eradication therapy. Then, the urine ACR was tested again, and the results indicated that, for all the follow-up of patients after *H. pylori* eradication, urine ACR declined significantly (Figure 2), that the urine ACR values decreased, respectively, by 40.4%, 41.4%, 46.7%, 63.6%, and 65.6%, whereas urine ACR levels in three of them were declined to normal.

## Discussion

*H. pylori* infection will increase the risk of gastrointestinal diseases, even gastric cancer [13]. Therefore, it is indispensable to monitor the *H. pylori* infection status of population especially the patients with gastrointestinal diseases. The *H. pylori* infection rate in patients with peptic ulcer is much higher than that in healthy controls, which is suggestive of the role of *H. pylori* infection in the pathogenesis of peptic ulcer disease [14]. This was demonstrated in our study (Table 1). It is reported that *H. pylori* infection is also associated with other chronic diseases, such as cardiovascular diseases, respiratory diseases, hematological diseases, metabolic dysfunction diseases, etc. [2]. Therefore, there is no doubt that the occurrence of chronic diseases in patients with peptic ulcer and *H. pylori* infection should not be neglected.

Besides, *H. pylori* infection is probably involved into chronic kidney damage according to basic research [8] and clinical evidence [15]. Lin et al. [15] reported the association between *H. pylori* infection and a subsequent risk of end-stage renal disease (ESRD). One explanation is that the systemic inflammation might play a role in the relationship between *H. pylori* infection and chronic kidney damage. It has been reported that chronic inflammation induced by *H. pylori* may be one of the major causes to renal diseases [9,16]. Increasing inflammatory cytokine level leads to the damage of vascular endothelial structure in kidney, resulting in albumin escaping from the kidneys into the urine. *H. pylori* infection induces the expression of inflammatory cytokines, chemokines, growth factors, etc., causing an inflammatory microenvironment. In addition, *H. pylori* infection induces the release of cytokines and vascular active substances, such as C-reactive protein (CRP), tumor necrosis factor alpha (TNF-α), interleukin 1 (IL-1), interleukin 6 (IL-6), interleukin 8 (IL-8), heat shock protein (HSP), arising local and systemic immune responses, which aggravates microvascular damage [16]. hs-CRP is highly expressed in patients with *H. pylori* infection in gastric mucosa [17]. Due to increasing urinary albumin excretion rate, a high level of CRP is regarded as one of the key risk factors to chronic kidney damage [18–20]. TNF-α can induce the expression of vascular endothelial cell adhesion molecules, which result in the proliferation of glomerular mesangial cells [21]. Thus, *H. pylori* infection might lead to chronic kidney damage or accelerated loss of kidney function through arousing systemic inflammation.

Although emerging evidence showed the association of *H. pylori* infection and chronic kidney disease [22,23], several studies reported no association [24,25]. To date, the association of *H. pylori* infection with chronic kidney disease has been controversial. In our work, we found the association of *H. pylori* infection and chronic kidney disease was only present in patients with peptic ulcer rather than matched healthy controls. As depicted in Table 2, there is no difference on the abnormal ACR rate or serum underglycosylated IgA1 level between *H. pylori* infected group and uninfected group in healthy control, which means *H. pylori* infection is not positively correlated to kidney damage in healthy controls. However, in patients with peptic ulcer, the abnormal rate data show a significant difference between *H. pylori*-positive group and *H. pylori*-negative group, indicating *H. pylori* infection is positively correlated to kidney damage in patients with peptic ulcer (Table 3). *H. pylori* infection always leads to a range of gastrointestinal symptoms in patients, accompanying the increase of gastrin and delayed gastric emptying. Severe gastrointestinal symptoms or atrophic gastritis potentially cause folate and vitamin B12 intake decrease, homocysteine increase, decline of adenylate, promoting oxidative stress, and accumulating oxygen free radicals [26], which will result in the lesion of vascular endothelium, ultimately triggering the kidney damage [27]. Besides, Gong et al. [28] confirm that patients’ chronic *H. pylori* infection elevates low-density lipoprotein level in patients and chronic or acute *H. pylori* infection will alter the serum levels of white blood cells, CD4^+^ T cells, low-density lipoprotein, high-density lipoprotein, which are the warnings of renal diseases. Therefore, it is necessary for the patients with peptic ulcer to monitor the status of *H. pylori* infection for protecting from kidney damage or related renal diseases.

However, in this present study, there is indeed no correlation between the severity of *H. pylori* infection and the levels of urine ACR (Table 4). There are two possible reasons. The first one is *H. pylori* has different strains that cause varying degrees of virulence to hosts [29]. The second one is the immune regulatory capacity of hosts responding to the same strains shows difference [30]. We did not examine the infected *H. pylori* strains of patients neither the virulence factors of *H. pylori*, such as cagA, vacA, dupA, iceA, oipA, and babA; therefore, further study is required to explain this phenomenon.

*H. pylori* antigens existed in the glomeruli of membranous nephropathy patients [31], and a significantly higher *H. pylori* infection rate in membranous nephropathy patients than that in control group [32]. Further studies on eradication of *H. pylori* successfully reduced proteinuria in patients with membranous nephropathy [32,33], patients suffering from dyspeptic complaints [9] and type 2 diabetic patients [34]. Similarly, our follow-up research on the *H. pylori* eradication showed that the urine ACR dramatically decrease after successful eradication by routine treatment (Figure 2), which enhances our conclusion that *H. pylori* infection should be established as one of the key risk factors for kidney damage or renal diseases in patients with peptic ulcer. The results contribute the research on the relationship between *H. pylori* and kidney damage, and enable us to take *H. pylori* eradication into consideration when exerting therapy for the patients with gastrointestinal diseases.
